# Differential hypomethylation of the repetitive *Tol2/Alu*-rich sequences in the genome of *Bodianus* species (Labriformes, Labridae)

**DOI:** 10.3897/CompCytogen.v12i2.21830

**Published:** 2018-03-28

**Authors:** Clóvis C. Motta-Neto, André Marques, Gideão W.W.F. Costa, Marcelo B. Cioffi, Luiz A.C. Bertollo, Rodrigo X. Soares, Kátia C. Scortecci, Roberto F. Artoni, Wagner F. Molina

**Affiliations:** 1 Center of Biosciences, Department of Cellular Biology and Genetics, Federal University of Rio Grande do Norte, Natal, Brazil; 2 Laboratory of Plant Cytogenetics and Evolution, Department of Botany, Federal University of Pernambuco, Recife, Brazil; 3 Department of Genetics and Evolution, Federal University of São Carlos, São Paulo, Brazil; 4 Department of Structural and Molecular Biology and Genetics, State University of Ponta Grossa, Ponta Grossa, Brazil

**Keywords:** Fish cytogenetics, Methylation, BOD region, pseudo-NORs, Mobile elements, Repetitive DNA

## Abstract

Representatives of the order Labriformes show karyotypes of extreme conservatism together with others with high chromosomal diversification. However, the cytological characterization of epigenetic modifications remains unknown for the majority of the species. In the family Labridae, the most abundant fishes on tropical reefs, the genomes of the genus *Bodianus* Bloch, 1790 have been characterized by the occurrence of a peculiar chromosomal region, here denominated BOD. This region is exceptionally decondensed, heterochromatic, argentophilic, GC-neutral and, in contrast to classical secondary constrictions, shows no signals of hybridization with 18S rDNA probes. In order to characterize the BOD region, the methylation pattern, the distribution of *Alu* and *Tol*2 retrotransposons and of 18S and 5S rDNA sites, respectively, were analyzed by Fluorescence *In Situ* Hybridization (FISH) on metaphase chromosomes of two *Bodianus* species, *B. insularis* Gomon & Lubbock, 1980 and *B. pulchellus* (Poey, 1860). Immunolocalization of the 5-methylcytosine revealed hypermethylated chromosomal regions, dispersed along the entire length of the chromosomes of both species, while the BOD regions exhibited a hypomethylated pattern. Hypomethylation of the BOD region is associated with the precise co-location of *Tol*2 and *Alu* elements, suggesting their active participation in the regulatory epigenetic process. This evidence underscores a probable differential methylation action during the cell cycle, as well as the role of *Tol*2/*Alu* elements in functional processes of fish genomes.

## Introduction

Genomes of some representatives of Labriformes families carry preferential chromosomal rearrangements (Sena and Molina 2007; Molina et al. 2014; Almeida et al. 2017), and singular regional DNA organization (Molina et al. 2012; Amorim et al. 2016). Labridae, the fifth largest marine fish family, with approximately 600 species, displays remarkable ecological and evolutionary diversification (Parenti and Randall 2000). Its phylogeny, where the relationships of the highest categories have been better recognized, is a long-standing and widely discussed problem (Westneat and Alfaro 2005). Particular evolutionary trends in karyotype differentiation, such as pericentric inversions and centric fusions, occur among tribes of this family (Molina and Galetti 2004, Sena and Molina 2007, Molina et al. 2014, Almeida et al. 2017). Indeed, while some groups show karyotype conservatism (Sena and Molina 2007), others possess karyotypes modeled by pericentric inversions, e.g. in the tribe Hypsigenyini and, particularly, the species of the genus *Bodianus* Bloch, 1790 (Molina et al. 2012).

The representatives of the tribe Hypsigenyini exhibit relatively symmetrical karyotypes, with 2n = 48 and high fundamental number (NF) values as compared to other ones (Arai 2011). Some Atlantic species, such as *Bodianus rufus* (Linnaeus, 1758), *B. pulchellus* (Poey, 1860) and *B. insularis* Gomon & Lubbock, 1980, have been analyzed in detail, and phylogenetically shared particular chromosomal regions have been identified. These regions, located at the p arms of the second subtelocentric chromosome pair, were characterized as exceptionally decondensed, heterochromatic and argentophilic, suggesting the presence of rDNA sites. However, these regions are neither GC-rich, nor do they display hybridization signals with 18S rDNA probes, indicating the presence of distinct repetitive sequences with unusual organization (Molina et al. 2012).

Molecular analyses have significantly widened the knowledge of the genomic organization and epigenetic modeling of the chromatin, particularly with respect to histone modifications of the euchromatin and heterochromatin (Fuchs et al. 2006). DNA methylation is catalyzed by a conserved class of DNA methyltransferases (Dnmt’s) broadly present in protists, fungi, plants and animal genomes (Craig and Bickmore 1994; Dyachenko et al. 2010). Islets of CpG dinucleotides (C-phosphate-G, on the fifth carbon) are correlated with 5-methylcytosine content (5 mC) (Vanyushin et al. 1973), which indicates hyper- and hypomethylation patterns in the chromatin related to gene regulation (Baylin et al. 1991; Almeida et al. 1993; Feinberg 1993; Barbin et al. 1994).

Although the knowledge of the methylation patterns is growing among vertebrates, it is still restricted in fishes, especially in relation to repetitive DNA regions (transcriptional and non-transcriptional), which are apparently limited to the heterochromatic regions and sex chromosomes (Schmid et al. 2016). Repetitive sequences have been the target of intense investigation in several fish groups (Vicari et al. 2008; Cioffi et al. 2010b; Costa et al. 2014, 2016; Barbosa et al. 2015), showing extreme complexity in some species (Costa et al. 2015). In this context, probable synergic or antagonistic interactions between collocated distinct sequences still need to be clarified.

In this study, we analyzed the DNA methylation pattern in the metaphase chromosomes of *B. pulchellus* and *B. insularis*, phylogenetically very close species (Gomon 2006), especially in the exclusive decondensed region (Ag+/CMA0/C+), here referred as BOD, in allusion to genus *Bodianus*. The data were compared with the structural patterns of the chromosomes, identified by the 18S and 5S rDNAs and the transposable elements *Tol*2 and *Alu* mapping using FISH.

## Methods

### Individuals, collection sites, chromosome preparation and bandings

Individuals of *Bodianus pulchellus* (n = 6, all immature individuals) from Bahia State (12°58'20"S, 38°31'05"W), on the northeastern Brazilian coast, and *B. insularis* (n = 5, 2 males and 3 immature individuals) from São Pedro and Paulo Archipelago (0°55'19"N, 29°21'44"W), were used in cytogenetic analyses. The individuals were collected under authorization provided by the Chico Mendes Institute of Biodiversity Conservation (ICMBIO/SISBIO) (license #02001.001902/06-82) and all experimental procedures followed the rules of the Animal Ethics Committee of the Federal University of Rio Grande do Norte (protocol 044/2015).

Mitosis stimulation followed the protocols developed by Molina (2001) and Molina et al. (2010). Mitotic chromosomes were obtained by means of the *in vitro* interruption of the cell cycle (Gold et al. 1990). An amount of 150μl of cell suspension was dropped onto a wet slide covered by a film of distilled water, heated to 60 °C and dried at room temperature. The Ag-NOR (Nucleolus Organizer Regions) sites and the extra nuclear argentophilic regions were identified according to Howell and Black (1980).

### FISH and immunostaining of methylated DNA

FISH was performed according to Pinkel et al. (1986). The 5S and 18S rDNA sequences were detected by double-color FISH analyses. Both ribosomal sequences were isolated from the *Hoplias malabaricus* (Bloch, 1794) (Teleostei, Characiformes) genome. The 5S rDNA included 120 base pairs (bp) of the 5S rRNA gene and 200bp from the non-transcribed spacer (NTS) (Martins et al. 2006). The 18S rDNA probes corresponded to a 1400bp segment from the 18S rRNA gene, obtained through PCR of the nuclear DNA (Cioffi et al. 2010a). The 5S rDNA probes were labeled with biotin-14-dATP by nick translation according to the manufacturer’s recommendations (BioNick Labeling System; Invitrogen, San Diego, CA, USA). The 18S rDNA was labeled by nick translation with Digoxigenin-11-dUTP, in line with the manufacturer’s recommendations (Roche, Mannheim, Germany). The *Tol*2 transposon probes were obtained by PCR of the nuclear DNA of *Rachycentron canadum* using the primers Tol2-5F 5'-CTG TCA CTC TGA TGA AAC AG-3' and Tol2-5R 5'-CTT TGA CCT TAG GTT TGG GC-3' (Kawakami and Shima 1999). The probes were labeled with Digoxigenin-11-dUTP by nick translation following the manufacturer’s recommendations (Roche, Mannheim, Germany). The (TTAGGG)n sequences were mapped by FISH using Telomere PNA FISH Kit/FITC according to manufacturer’s instructions (Dako Citomation). The *Alu* transposon probes were obtained by PCR of the genomic DNA of *Rachycentron canadum* (Linnaeus, 1766) using the primers Alu CL1 5’-TCC CAA AGT GCT GGG ATT ACA G-3’ and Alu CL2 5’-CTG CAC TC AGC CTG GG-3’ (Lengauer et al. 1992), and were labeled with Digoxigenin-11-dUTP by nick translation following the manufacturer’s recommendations (Roche, Mannheim, Germany). The chromosomes were counter-stained with Vectashield/DAPI (1.5mg/ml) (Vector) and photographed with an Olympus BX50 epifluorescence microscope coupled to an Olympus DP73 digital camera, using CELLSENS software (Olympus).

The DNA methylation patterns in the metaphase chromosomes were detected through binding analysis of the monoclonal antibody to 5-methylcytosine. Indirect immunodetection of the methylated DNA was conducted according to Marques et al. (2011). The slides were treated with 20 mg/ml RNAse (Invitrogen) diluted 1:200 in 2XSSC for one hour, followed by exposure to 1mg/ml pepsin (1:100) in 0.01 N HCl (100μl per slide) for 20 minutes. They were then denatured in 70% formamide for 3 min at 75 °C and blocked with 3 % BSA diluted in 1X PBS with 0.1 % Tween 20, for 30 minutes at 37 °C and incubated with the mouse-anti-5-methylcytosine primary antibody (Eurogentec) in 1 % BSA/1X PBS (1:100) overnight at 4 °C. The 5 mC was detected using anti-mouse-FITC diluted (1:200) in 1 % BSA/1X PBS for 1 hour at 37 °C. Finally, the slides were washed in 1X PBS, mounted with DAPI/Vectashield antifading (Vector Laboratories) and analyzed by fluorescence microscopy under a Leica DMBL photomicroscope (Leica Microsystems) equipped with a CCD Cohu camera (CohuHD Costar), using QFISH software. Composition of the image with the hybridization signals was done with Photoshop CS5 (Adobe) software.

The chromosomes were categorized as metacentric (m), submetacentric (sm), subtelocentric (st) and acrocentric (a), according to Levan et al. (1964) and arranged in descending order of size. A single ideogram for both species was constructed in order to highlight the repetitive sequences and the methylation patterns identified by 5 mC.

## Results

**Figure 1.**
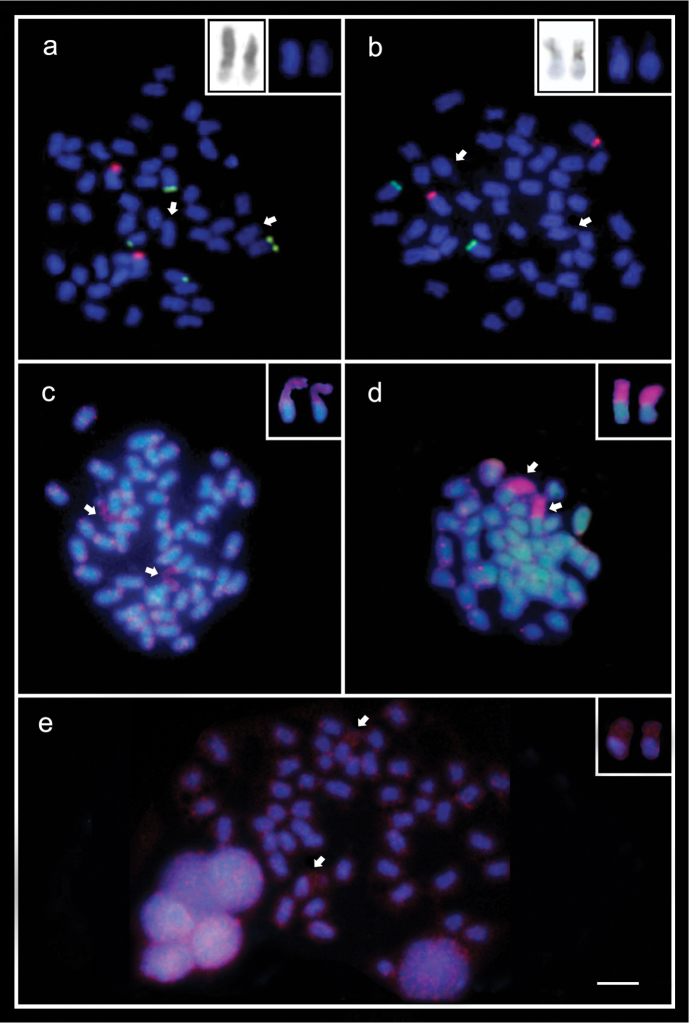
Metaphase chromosomes of the species *B. insularis* (**a, c, e**) and *B. pulchellus* (**b, d**); the chromosome pairs bearing the BOD region are identified with arrows and highlighted in the boxes **a, b** 18S (red signals) and 5S rDNA FISH (green signals). In the boxes, the argentophilic pattern showed in the BOD regions and the DAPI staining pattern, respectively **c, d** Distribution of the *Tol*2 element in the chromosomes. An accumulation of *Tol*2 sequences is perceptible in the BOD regions **e** - Distribution of the *Alu* transposable element on the chromosomes of *B. insularis*. Scale bar: 5μm.

**Figure 2.**
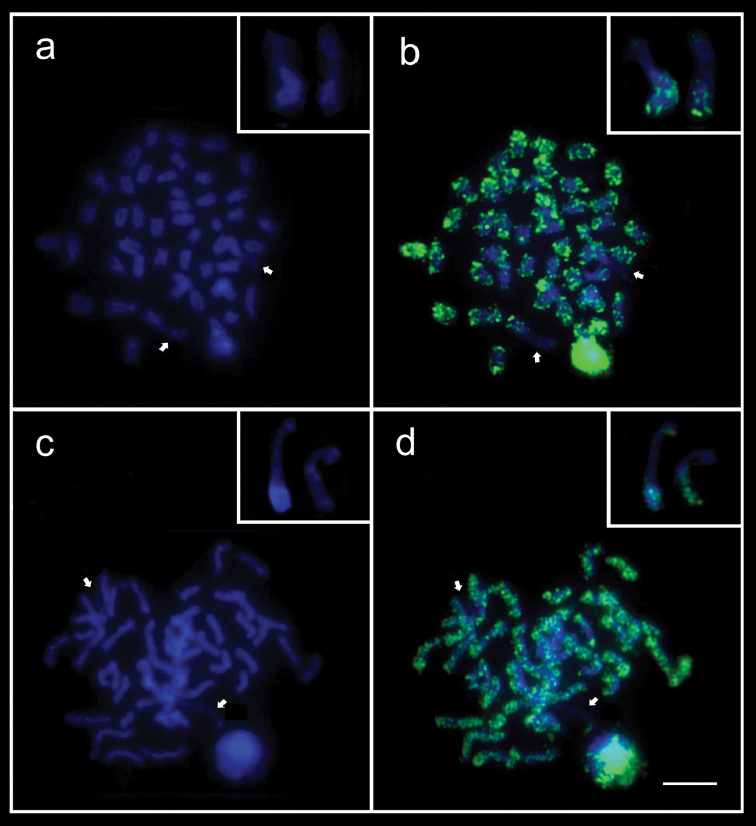
Metaphase chromosomes of the species *Bodianus pulchellus* (**a, b**) and *Bodianus insularis* (**c, d**) after DAPI staining (left) and sequential immunodetection of methylated sites with the monoclonal antibody 5mC (right). The chromosome pairs bearing the BOD region are denoted with arrows and highlighted in the boxes. Scale bar: 5μm.

*Bodianus pulchellus* and *B. insularis* have diploid chromosome number 2n=48 and identical karyotypes, composed of 4m+12sm+14st+18a chromosomes, NF value 78. As previously described for these species (Molina et al. 2012), the 10^th^ subtelocentric pair exhibited an extensive decondensed terminal region that could reach up to four times the size of the largest chromosome pair (Figs 1, 2) – the BOD region.

Ag-NOR sites were located in the terminal region of the pair No. 9 in karyotypes of both species (Fig. 1a, b). These sites and the BOD region were also argentophilic (Fig. 1a, b; highlighted), as in previous descriptions (Molina et al. 2012).

Double-FISH with 5S and 18S rDNA probes revealed a non-syntenic location for these ribosomal sites. The 18S rDNA sites were exclusively located in the terminal regions on the p arms of the pair No. 9, corresponding with the Ag-NOR signals. No hybridization signals were detected in the BOD regions of both species (Fig. 1a, b). On the other hand, 5S rRNA genes were located in the terminal regions on the q arms of the pair No. 16 in both species, and an extra pericentromeric site on the p arms of the pair No. 19, only in *B. insularis* (Fig. 1a, b).

The hybridization with the transposable element *Alu* was only performed in *B. insularis*, while *Tol*2 mapping was performed in both species. These sequences exhibited a similar distribution pattern in the chromosomes preferentially located in the terminal regions of the chromosomes and particularly accumulated in the BOD one (Fig. 1c–e; highlighted).

The hybridization signals with (TTAGGG)_n_ probes were variable, with the majority having the same size, besides some chromosomes showed no detectable signals (data not shown). Immunostaining with 5 mC revealed that most metaphase chromosomes of the two species were hypermethylated (Fig. 2b, d). By contrast, the BOD regions were distinctly hypomethylated, as well as the centromeric regions of the majority of chromosomal pairs (Fig. 2).

**Figure 3.**
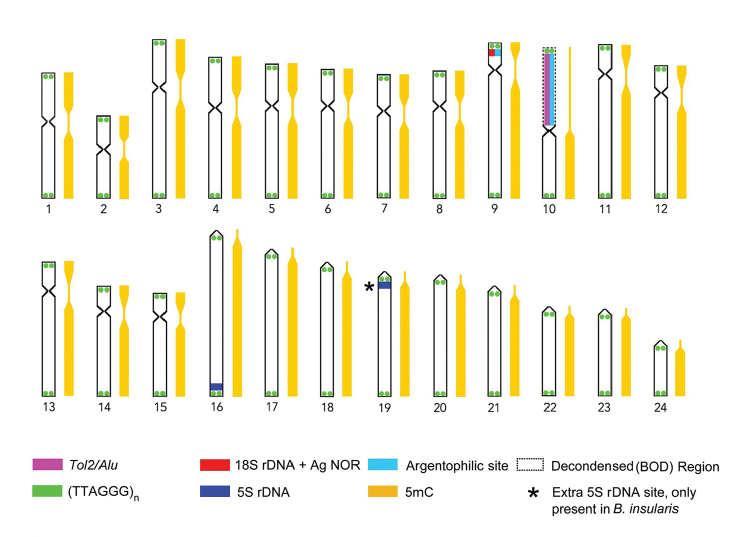
Ideogram showing distribution of repetitive sequences and the methylation pattern in the metaphase chromosomes of *B. insularis* and *B. pulchellus*, a dashed line highlights the decondensed BOD region.

All results were summarized in the ideogram of the Figure 3 below.

## Discussion

### Structural chromosome characteristics of *Bodianus* species

The heterochromatic regions of fish genomes have been the target of intense investigation. Although displaying variations in the amount and distribution on chromosomes, heterochromatin can harbor a diversified panel of collocated sequences, whereby the effects of this interaction under gene regulation and dispersion pattern need to be better investigated (Costa et al. 2015). Functional biases could derive from the joint distribution of multigene families, or from their association with other repetitive sequences, constituting adaptive aspects and implying maintenance and dispersion in the chromosomes (Costa et al. 2016).

Argentophilic decondensed regions in vertebrates are often related to NOR sites (Árnason 1981; Schmid et al. 1982; Birstein 1984; Supanuam et al. 2012). Previous cytogenetic studies identified an intriguing chromosomal region on homeologous pairs in *B. pulchellus*, *B. insularis* and *B. rufus* (Molina et al. 2012). This region, now identified as BOD one, exhibits a high decondensed structure and a heterochromatic, GC-neutral and argentophilic constitution, but that does not exhibit any hybridization signals with 18S rDNA probes. The sharing of this particular set of constitutive and functional characteristics indicates that the origin of the BOD region precedes the phyletic diversification of those species (Molina et al. 2012), representing a very favorable *sui generis* condition for the study of the complexity of repetitive DNA arrangements in fishes.

In several vertebrate species, including fishes, argentophilic sites not associated with rDNA sites, known as pseudo-NORs, were already described (Ozouf-Costaz et al. 1997; Pisano et al. 2000; Caputo et al. 2002; Dobigny et al. 2002; Gromicho et al. 2005; Cabrero and Camacho 2008). Structurally, the BOD regions have similarities with pseudo-NORs that are tandem arrays of a heterologous DNA sequences. In some species, the pseudo-NORs do not exhibit promoter sequences and have high affinity for the upstream binding factor (UBF), a DNA binding protein and component of the Pol I transcription machinery which binds extensively across the rDNA repeat *in vivo* (Prieto and McStay 2008). The formation of pseudo-NORs is associated to a special class of multigene families, like histones and ribosomal genes, from both protein- and non-protein-coding with capacity of translocation known as orphons (Childs et al. 1981, Cabrero and Camacho 2008).

Pseudo-NORs used to mimic real NORs in several aspects, as they can remain decondensed during mitosis when the transcription is inactivated and the nucleolus is broken down, forming novel silver positive secondary constrictions (Mais et al. 2005). UBF can displace histone H1 from histone octamers *in vitro* (Kermekchiev et al. 1997), thereby promoting the chromatin decompaction. Additionally, Ag-positive loci can be the result of the presence of residual acidic proteins with affinity for silver, reacting with this compound (Dobigny et al. 2002). In fact, pseudo-NORs are reactive to silver staining despite their transcriptional silence (Mais et al. 2005). The typical decondensation observed in secondary constrictions should be promoted by the action of binding argyrophilic proteins that prevent the full condensation of that region (Prieto and McStay 2008). These elements could explain the argentophilic and decondensed nature of the chromatin present in the BOD region.

Representatives of *Bodianus* display karyotypes with a larger number of biarmed chromosomes when compared to those of other Labridae genera (Sena and Molina 2007). This is a synapomorphic pattern and indicates intense structural chromosome reorganization in this clade. However, several common characteristics, such as the presence of a single 18S rDNA site and the BOD region, could indicate a lower level of diversification among the youngest branches of this group. In fact, the presence of a single chromosome pair bearing 18S rDNA sites represent the most frequent pattern found in fishes (Gornung 2013) as well as in several perciform groups (Motta-Neto et al. 2012; Costa et al. 2016). On the other hand, 5S rDNA sites show a more diversified pattern, being present on a single chromosome pair in *B. pulchellus* but on two pairs in *B. insularis*. The monitoring of ribosomal genes in chromosomes in a phylogenetic perspective makes it possible to identify the sequential patterns of change or synteny maintenance over time (Affonso et al. 2014; Fernandes et al. 2015; Costa et al. 2016), especially in conserved karyotypes, such as in *Bodianus*.

In genomes of some fish species, a high chromosome dynamism has been identified for *Tol*2 elements, which can be situated in different genomic regions (Koga and Hori 1999), or be preferentially concentrated and collocated with 18S rDNA sites (Costa et al. 2013). Therefore, the presence of structural and functional characteristics of the BOD region, typical of pseudo-NORs, may indicate that these regions were originally repositories of rDNA. Indeed, *Alu* and *Tol*2 elements exhibit a remarkable accumulation in the BOD regions. Transposable elements are transposed by a cut-and-paste mechanism, involving their excision and insertion elsewhere in the chromatin. Additionally, the spreading of transposons can be concatenated with the capacity of the orphons translocation through the genome via dispersion and magnification of minor loci consisting of a few rDNA copies (Dubcovsky and Dvorak 1995) as observed in *Aegilops speltoides* Tausch (Flaksberger 1935) (Raskina et al. 2004). If the excision process of transposons is excessive, it may affect the function of a particular gene, making it functionally unstable, requiring only that the transposon insertion occurs within or very close to the gene (Lippman et al. 2004), as observed in this study.

*Alu* elements concentrate huge amounts of CpG islands that are genomic regions that contain a high frequency of CpG dinucleotides, commonly representing promoters, which are usually located in GC dense regions. CpG islands tend to be hypomethylated allowing an open chromatin organization and facilitating neighboring gene expression (Jones and Baylin 2002; Gu et al. 2016). On the other hand, *Alu* sequences are punctuated by multiple CpG domains, many of which overlapping with known protein binding sites (Rowold and Herrera 2000), possibly the same aforementioned argyrophilic binding proteins which keep the chromatin decondensed and consequently opened. Thus, the marked occurrence of *Alu* and *Tol*2 elements in the BOD regions could have significantly interfered in the ribosomal gene functionality, causing a pseudogenization process.

### Differential methylation in *Bodianus* metaphase chromosomes

Despite the fact that a significant part of the genome of some organisms is composed of repetitive DNA sequences, their origins, dispersion and functional interaction remain largely unknown (Biémont and Vieira 2006). In this context, methylation patterns help us understand the functional aspects of the genome. DNA methylation is an important epigenetic modification in the genome of vertebrates, where only small fractions of it are hypomethylated (Nakamura et al. 2014). An overview of methylation in the vertebrate genome indicates that more basal groups such as fish and amphibians have higher methylation levels than reptiles, mammals and birds and is inversely related to body temperature (Vanyushin et al. 1973; Jabbari et al. 1997; Varriale and Bernardi 2006a, b). Despite the occurrence of chromosomal rearrangements associated with DNA methylation, this process may suppress homologous recombination, enabling genomes rich in repeats to remain relatively stable (Colot and Rossignol 1999).

The immunolocalization of 5-methylcytosine in the metaphase chromosomes of the two *Bodianus* species revealed a primarily hypermethylated pattern, despite the striking contrast observed in the BOD and the centromeric regions, both notably hypomethylated. In general, centromeric regions exhibit particular epigenetic characteristics, including DNA hypermethylation. The presence of hypomethylated regions in the centromeres of some chromosome pairs of the *Bodianus* species demonstrates an uncommon and likely functional condition of these regions, which are closely associated with the chromosome segregation process.

DNA methylation is considered a controlling mechanism of gene expression, including the ribosomal ones (Ferraro and Lavia 1983; Ferraro and Prantera 1988). Indeed, there is an inverse correlation between DNA methylation and the transcriptional activity of several eukaryotic genes (Kanungo 1994), as well as nucleolar size and the number of rDNA loci sites (Bacalini et al. 2014). In mammals, there is a strong relation between states of DNA methylation and gene silencing (Eden et al. 1994). On the other hand, in invertebrates, the origins and meaning of methylation patterns show, in some cases, the absence of a correlation between methylation and gene expression (Tweedie et al. 1997). In more basal fish groups, GC-rich heterochromatins, which are frequently related to NOR regions (Gornung 2013), are highly methylated in the germ line, but to a lesser degree in somatic chromosomes (Covelo-Soto et al. 2014). The hypomethylation patterns of repetitive and ribosomal DNA classes can lead to chromatin decondensation (Carvalho et al. 2000; Jones and Baylin 2002), as demonstrated here for the BOD regions.

In some Perciformes species, *Tol*2 elements are distributed along the chromosomes and distinctly associated with 18S rDNA sequences (Costa et al. 2013). In *Bodianus*, both the accumulation of these transposons in the BOD regions as well their hypomethylated nature, are prominent. It has been reported that the methylation process plays a protective role against invasive DNAs or transposable elements (Yoder et al. 1997; Doerfler 1991) and is a key mechanism in gene regulation and expression (Finnegan et al. 1998; Heslop-Harrison 2000; Attwood et al. 2002). Indeed, the transposable sequences in the human genome are highly methylated (Kricker et al. 1992). Fishes have shown hypermethylated regions confined to constitutive heterochromatin, particularly in heteromorphic sex chromosomes, demonstrating that several hypermethylated regions are co-localized with repetitive elements (Schmid et al. 2016).

It is known that DNA methylation may limit the dispersion of various transposable elements in a number of genomes (Scortecci et al. 1997; Miura et al. 2001; Iida et al. 2006). However, this condition does not occur in the BOD regions. If methylation inhibits the dispersion of transposable elements, why is the BOD region, extremely rich in *Alu* and *Tol*2 elements are not methylated? The answer may be related to the following considerations: (1) *Alu* elements appear to be preferentially located in GC-rich genomic isochores (Deininger 2006), explaining the accumulation of this transposable element in the 18S rDNA sites; (2) CpG islands, strongly present in *Alu* elements, are hypomethylated as a response to an overlapping between the CpG domains and the argyrophilic proteins binding sites, which prevent the full condensation of the heterochromatin through the displacement of the histone H1 from the histone octamers. This way, the open and decondensed heterochromatin may offer favorable conditions for the accumulation of the *Tol*2 retrotransposon in such region; (3) the epigenetic action promoted by the excessive excision of transposons inserted within or very close to the gene in the BOD region, affects its function and makes it functionally unstable. Therefore, novel NORs are formed in other chromosomal locations by the transposons spreading, associated with the translocation of orphons and the magnification of minor rDNA loci. Evidence suggests that in some fish species, such as *Oryzias latipes* (Temminck and Schlegel 1846), the *Tol*2 element, underwent a rapid expansion in the past, but acquired interactive control mechanisms (Iida et al. 2006). Therefore, in the same way compensatory evolutionary mechanisms may have been fixed in the *Bodianus* BOD region, thereby controlling the activity and dispersion of *Tol*2 and *Alu* elements. The delimitation of a preferential reservoir for these transposable elements in the BOD region would therefore constitute effective protection for genes allocated to the other chromosomes of the karyotype.

## Conclusion

DNA methylation is one of the epigenetic processes that has modulated the molecular evolution of life, but its influence in karyotype evolution and interaction in the structural chromosome regions are little known, especially for fish species. The use of monoclonal antibodies in cytogenetic study of *Bodianus* species provided an overview of the methylation pattern of metaphase chromosomes, with sufficient resolution to characterize the peculiar BOD regions. The complex composition of the BOD chromatin suggests that it is a pseudo-NOR containing a relict sequence of an ancestor rDNA. The DNA organization of such region provided evidence of its functional dynamics, possibly in the transcriptional control of *Tol*2 and *Alu* elements. In this sense, the methylation process, associated with the dispersion control of the transposable elements, may have played a particular active role in the evolutionary process of *Bodianus* species.
